# Functional Folate Receptor Alpha Is Elevated in the Blood of Ovarian Cancer Patients

**DOI:** 10.1371/journal.pone.0006292

**Published:** 2009-07-20

**Authors:** Eati Basal, Guiti Z. Eghbali-Fatourechi, Kimberly R. Kalli, Lynn C. Hartmann, Karin M. Goodman, Ellen L. Goode, Barton A. Kamen, Philip S. Low, Keith L. Knutson

**Affiliations:** 1 Department of Immunology, Mayo Clinic, Rochester, Minnesota, United States of America; 2 Department of Oncology, Mayo Clinic, Rochester, Minnesota, United States of America; 3 Department of Health Sciences Research, Mayo Clinic, Rochester, Minnesota, United States of America; 4 The Leukemia and Lymphoma Society, White Plains, New York, United States of America; 5 Department of Chemistry, Purdue University, West Lafayette, Indiana, United States of America; Bauer Research Foundation, United States of America

## Abstract

**Background:**

Despite low incidence, ovarian cancer is the fifth leading cause of cancer deaths and it has the highest mortality rate of all gynecologic malignancies among US women. The mortality rate would be reduced with an early detection marker. The folate receptor alpha (FRα) is one logical choice for a biomarker because of its prevalent overexpression in ovarian cancer and its exclusive expression in only a few normal tissues. In prior work, it was observed that patients with ovarian cancer had elevated serum levels of a protein that bound to a FRα-specific monoclonal antibody relative to healthy individuals. However, it was not shown that the protein detected was intact functional FRα. In the current study, the goal was to determine whether ovarian cancer patients (n = 30) had elevated serum levels of a fully functional intact FRα compared to matched healthy controls (n = 30).

**Methodology/Principal Findings:**

FRα levels in serum were analyzed by two methods, immunoblotting analysis and a radiolabeled folic acid-based microfiltration binding assay. Using the immunoassay, we observed that levels of FRα were higher in serum of ovarian cancer patients as compared to controls. Similar results were also observed using the microfiltration binding assay, which showed that the circulating FRα is functional. Importantly, we also found that the levels of FRα were comparable between early and advanced stage patients.

**Conclusions:**

Our results demonstrate that ovarian cancer patients have elevated levels of functional intact FRα. These findings support the potential use of circulating FRα as a biomarker of early ovarian cancer.

## Introduction

Ovarian cancer is the fifth leading cause of cancer death and has the highest mortality rate of all gynecologic malignancies among US women with an estimated 21,650 new cases and an anticipated >15,500 deaths in 2008 [1]. The majority of patients with ovarian cancer present with advanced disease (stages III–IV) and have a 5-year survival of typically <30%. The fact that survival of patients with stages I–II disease ranges from 60%–90%, depending on tumor grade [1], [2] suggests the potential for a high cure rate with earlier disease detection. Since physical symptoms are absent in early stages of ovarian cancer, efforts are being made to develop assays for blood or tissue biomarkers.

Although the serum CA-125, an ovarian cell surface glycoprotein (i.e. a mucin) of unknown biological significance, is elevated in >80% of patients with advanced epithelial ovarian cancer, this marker has a positive predictive value of <10% in early stage disease [3]. Furthermore, CA-125 is also associated with various non-malignant conditions, such as pregnancy, endometriosis, adenomyosis, uterine fibroids, pelvic inflammatory disease, menstruation, and benign ovarian cysts. CA-125 is also associated with other malignant conditions such as pancreatic, breast, lung, gastric, and colonic cancers [4]. Although the CA-125 is helpful in the follow-up of the patient's chemotherapy and in detecting early relapse in patients with already known ovarian cancers, it is not useful for identifying early stage disease [5].

The folate receptor α (FRα), a 38–40 kDa molecule, is a well characterized member of the folate receptor (FR) family with high affinity for folates. FRα is anchored to cell membranes through a glycosylphosphatidylinositol moiety and transports folates via an endocytic process [6]. FRα exhibits limited normal tissue distribution, with measurable expression restricted to the apical surfaces of a few epithelia, predominantly in the lung, kidney, and choroid plexus, but is overexpressed in a spectrum of solid tumors, including ovarian cancer, non small cell lung cancer, breast cancer, kidney cancer and in high-grade osteosarcoma [7]–[10]. This over-expression of high affinity FRα in some cancers may have arisen to meet cellular requirements for DNA synthesis and growth [11].

In addition to its cell surface localization, FRα can also be shed into the blood. Shedding was initially identified during the development of the folate radioligand binding assays which used purified cell-free FR as a binding protein. In these studies it was noted that there was often a large discrepancy in the assessment of total serum folate when assayed after heat extraction as compared to native serum [12]. When shed, FRα will bind circulating folate, since the affinity is in the nM range. Now it is well understood that shedding complicates the interpretation of serum folate assays and possibly masks the presence of functional receptor unless an acid treatment is employed to release the endogenous folate and allow FRα saturation with the radiolabeled folic acid. This discrepancy in folic acid levels between native and heat extracted serum led to the identification of circulating FRα in some samples of umbilical cord blood and in some of the maternal serum [13]. Importantly, FRα released from normal epithelial cells should not contribute to FRα levels in the serum, since the receptor is invariably localized to the apical surface of a polarized epithelium where its shedding will deliver the free receptor into a lumen that naturally drains from the body through its own orifice.

Recent studies suggest that FRα may also be shed into the blood from tumors, which offers an opportunity to access and measure it as a tumor marker of early stage cancer. The prevalent expression of FRα in ovarian cancer, among all stages, has stimulated interest in applying it as a therapeutic target and biomarker [14]. The goal of the current study was to determine if intact functional FRα is shed into the circulation and to evaluate its potential as a circulating biomarker. This goal is supported by prior work which showed that ovarian cancer patients have elevated levels of a serum protein that react with anti-FRα antibodies [15]. What remained to be answered was whether the antibodies detected whole FRα and whether the detected protein was a functional folate receptor (FR). Thus, in the present study, the levels of circulating FRα in the blood of patients with ovarian cancer were examined using a ^3^H-FA-based microfiltration assay which was developed to detect functional FRα. Additionally, an immunoblot assay was employed to confirm the presence of the specific whole FRα protein. As will be shown, ovarian cancer patients demonstrate elevated levels of FRα in the circulation supporting its potential use as a disease biomarker.

## Materials and Methods

### Ethics Statement

The Mayo Clinic IRB approved the study and written informed consent was obtained from all the subjects.

### Subjects

Each ovarian cancer case was first matched to a female control on blood draw within 6 months (all 30 cases matched successfully), and then age within 5 years. Twenty-nine cases were successfully matched. The 30th case was matched on blood draw within 6 months and age within 9 years to an eligible control. Eligible cases were patients, over age 20, with histologically confirmed primary epithelial ovarian cancer. A 40-mL (pre-operative) blood sample was collected, processed, aliquoted, and stored at −80°C as serum. The mean age (±SEM) of the age-matched healthy female donors and patients were 61±3 and 60.63±3 years respectively. Other patient and tumor characteristics are presented in Table 1.

**Table 1:** Tumor and patient characteristics.

Tumor histology	n (%)
Serous	18 (60)
Serous borderline	3 (10)
Endometrioid	3 (10)
Clear cell	1 (3)
Mucinous borderline	3 (10)
Mixed	1 (3)
Transitional cell	1 (3)
**Grade**	
1	2 (7)
2	2 (7)
3	15 (50)
4	6 (20)
**FIGO stage**	
Stage I	13 (43)
Stage II	2 (7)
Stage III	6 (20)
Stage IV	9 (30)
**Debulking status**	
Optimal	25 (83)
Sub-optimal	5 (17)
**FRα expression**	
High	27 (90)
Low	3 (10)

### Supplies and reagents

Folic acid was obtained from Alexis Biochemicals. Centrifuge filters (10,000 dalton cutoff) were purchased from Millipore (Burlington, MA). Tritiated folic acid [3,5,7,9-^3^H] sodium salt, (^3^H-FA,1 mCi/ml) was purchased from American Radiolabeled Chemicals (St. Louis, Missouri), and Ready-Safe liquid scintillation cocktail was from Beckman Coulter. Anti-FRα antibodies, MOv18/ZEL and F5753 were from Alexis Biochemicals (San Diego, CA) and US Biological (Swampscott, MA), respectively.

### Preparation of tumor cell lysates and cell culture supernatants as sources of FRα

KB cells are human nasopharyngeal epidermoid carcinoma cells that express high levels of FRα [16]–[18]. KB cells were cultured in media consisting of folate-free RPMI 1640, supplemented with 55 µM 2-mercaptoethanol, 2 mM L-glutamine, 1 mM sodium pyruvate, 10 mM HEPES buffer, 100 IU/ml penicillin, 100 µg/ml streptomycin and 10% fetal bovine serum. Supernatants were prepared by centrifugation of conditioned media at 4°C to remove unanchored cells. Aliquots of 4-day supernatants were stored at −80°C to be used as a source of non-cell associated FRα. Cell lysates were prepared from confluent KB monolayers by repeated freeze-thaw cycles followed by centrifugation to remove debris. For both supernatants and cell lysates, protein concentrations were determined by optical density. MCF-7 human breast cancer cells were used as a FRα−negative control and prepared similarly to KB cells. Lysates (5–10 µg of lysates) and supernatants (10–20 µL) were used for assay development and as controls.

### ^3^H-folic acid binding assay

Measurement of folate receptors in cases and controls was a variation of a previously described method [19]. Samples (10 µl) were loaded into the upper chamber of a pair of filtration tubes and diluted to 50 µl total with a solubilizing solution (50 mM Tris, pH 7.4, 150 mM NaCl, 25 mM *n*-octyl-β-D-glucopyranoside, 5 mM EDTA, and 0.02% sodium azide). The filtration tubes were centrifuged and the filters treated with 30 mM acetate solution (pH 3.0) to remove endogenous FA followed by centrifugation and two washes with the solubilizing solution. Then pairs of filtration tubes were incubated either with a 1000X cold FA in a binding solution (10 mM Na_2_P_4_, 1.8 mM KH_2_PO_4_, 500 mM NaCl, 2.7 mM KCl, pH 7.4, and 25 mM *n-*octyl-β-D-glucopyranoside) or with the binding solution alone, and samples were incubated 2 hours at room temperature and then centrifuged. Binding solution containing ^3^H-FA was then added to all samples followed by incubation overnight at 4°C with gentle agitation. The filters were centrifuged and washed with PBS containing 50 mM *n*-octyl-β-D-glucopyranoside to remove the unbound ^3^H-FA. Bound ^3^H-FA retained on the filters was removed with 100 µl PBS containing 4% Triton X-100 and transferred into a vial with liquid scintillation cocktail, and the activity was measured in a Beckman LS6000IC Scintillation Counter. Specific binding was determined by subtracting the activity in the presence of excess cold FA from the activity of the same sample without FA competition.

### Immunoprecipitation and immunoblot analysis of FRα

FRα in the serum was directly measured using immunoblot analysis of immunoprecipitates. Serum samples (100 µl), lysates (25 µg KB or MCF-7) or KB supernatants were pre-cleared two times with Protein G Sepharose and 5 µg each of monoclonal antibodies, MOv18/ZEL and F5753, were added to each sample and incubated for 1 hour at 4°C. Protein G plus Sepharose beads were then added and the mixture was allowed to rotate overnight at 4°C. The beads were then collected with centrifugation and washed with 600 µl of lysis buffer (20 mM Tris-HCl, pH 7.5, 150 mM NaCl, 1 mM Na_2_EDTA, 1 mM EGTA, 1% Triton, and protease inhibitors). The beads were decanted and boiled in Laemmli buffer. The proteins were resolved by SDS-PAGE under non-reducing conditions followed by blotting. The blots were probed with MOv18/ZEL antibody (5 µg/ml in 2% nonfat dry milk, 0.1% Tween 20 in TBS) followed by secondary anti-mouse IgG horseradish peroxidase (HRP) conjugated antibody (1∶5000 in 2% non fat dry milk in 0.1% Tween 20), and developed by enhanced chemiluminescence with Super Signal West Pico Chemiluminescent Substrate for 5 minutes (Pierce, Rockford, IL, USA). The blots were then exposed to X-ray film and band positions and band densities were calculated using a computerized image analysis system (UVP Biochem Imaging systems, Upland, CA).

### Statistical Analysis

The mean levels of circulating FRα, detected with either ligand-binding assay or immunoblotting analysis, between cases and control subjects were compared using the Wilcoxon matched pairs test. In some cases linear regression analysis was used to examine for statistically significant correlations. Proportions were tested using Fisher's exact test. In all cases, a two-tailed test was used and a p value of less than or equal to 0.05 was considered significant.

## Results

### Detection of FRα using microfiltration binding assays and immunoblotting

**Figure 1:**
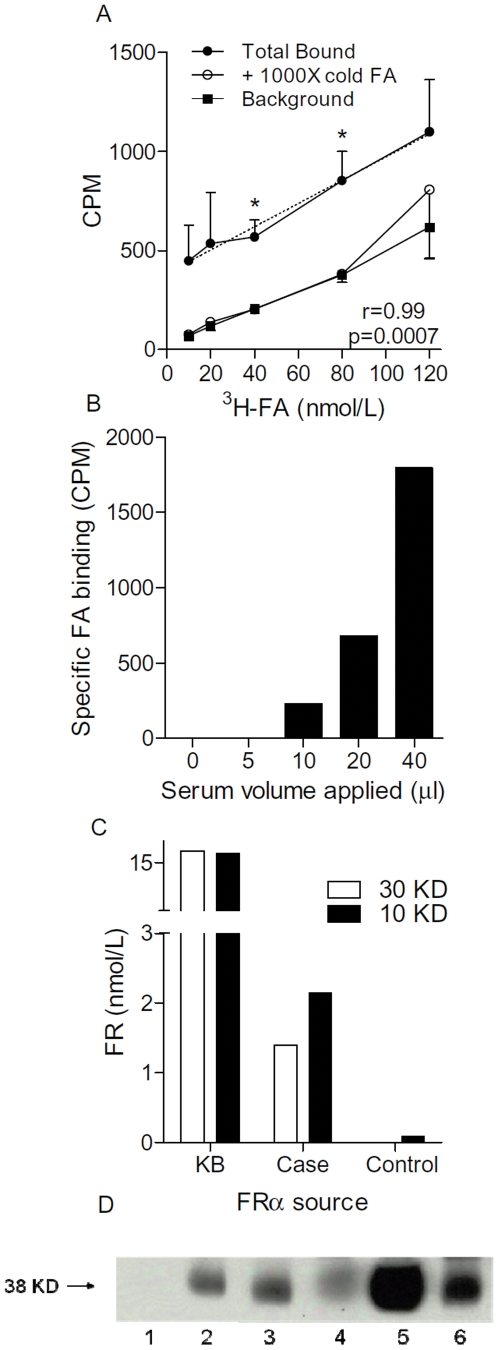
Detection of circulating FRα using microfiltration and immunoblotting. Panel A: Ten µl aliquots of KB culture supernatant was diluted to 50 µL with binding buffer that contained increasing concentrations of ^3^H-FA pulsed, without (Total bound) or with 1000X cold FA. After incubation, unbound FA was removed by microfiltration. Shown are the CPM retained by the microfilter. Also shown is Background which is binding in the absence of KB supernatants and without excess cold FA. *p<0.05. Each data point is the mean (±SE) of 3 replicates. The inset dotted line is the linear regression line of the total bound presented with the corresponding p and r values. Panel B shows that the amount of specific binding is dependent on the input levels. In this case increasing amounts of serum containing a KB protein spike were examined. This experiment is representative of 2 different experiments. Panel C shows the FA concentration that was bound in either KB supernatants or a representative serum sample from a case and control, comparing the 30 KD and 10 cutoff microfilters. Experiment is representative of two experiments. Panel D shows a representative example of immunoblot analysis of FRα. Arrow = M_r_ 38000 protein; KD = kilodalton; IP = immunoprecipitation. Lane 1, IP w no serum; lane 2, IP of KB lysate; lane 3, IP of precleared serum with KB lysate spike; lane 4. IP of serum alone, lane 5, KB lysate no IP; lane 6, IP of KB lysate no serum.

In initial efforts to measure levels of circulating FRα by ^3^H-FA binding studies, preliminary studies were done to determine the attributes of the microcentrifuge filtration assay. KB cell supernatant samples were incubated with increasing amounts of ^3^H-FA in the absence and presence of excess cold FA and then measured the recovery of bound ^3^H-FA. As shown in **Figure 1A**, between 10 and 120 nmol/L ^3^H-FA, the total binding increased in a linear fashion. When excess cold FA was added, the amount bound was reduced to background levels. The calculated FA concentrations over this range of (10–120 nmol/L) were consistent and none of the values were significantly different (not shown). The results show that the assay is able to measure FRα over a wide range of FA concentrations.

In order to determine the minimum amount of serum required, a normal control serum sample (100 µl) was spiked with KB cell lysate (5 µg) and different volumes (0–40 µL) were tested for binding ^3^H-FA. As shown in a representative example experiment in **Figure 1B**, the microfilter assay could detect FA binding in a volume as little as 10 µl. However above 20 µl of serum, it was found that the microfilters did not always readily drain. Thus, a standard volume of 10–20 µl was used for cases and controls.

Although prior work had used 30 KD cutoff filters for measuring FRα in tissue samples [19], there was a concern that some loss may have arisen as a consequence of a variation in pore size, resulting in the presence of some filter pores that would allow passage of the 38 KDa FRα. Therefore, tests were done comparing 30 KD and 10 KD cutoff filters. In general, there were only minor differences. For example, as shown in **Figure 1C**, there was no remarkable difference in the bound FA when using KB cell lysates of KB supernatants. In contrast, small increases were consistently observed in serum samples when using the 10 KD cutoff filters. Thus, the 10 KD cutoff was used to measure FRα in the sera of cases and controls.

In an effort to corroborate the microfiltration results, an immunoprecipitation and immunoblotting method was established. As shown in **Figure 1D**, FRα can be immunoprecipitated from sera (100 µl) and immunoblotted resulting in detection of the 38 KD FRα. Overall, these results show that the microfiltration assay can be used to detect FRα in small sera samples. Further, immunoblotting analysis reveals the presence of intact full-length FRα.

### Patients with untreated ovarian cancer have elevated mean levels of circulating FRα as compared to matched controls

**Figure 2:**
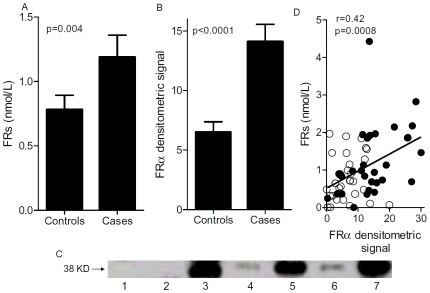
Patients with ovarian cancer have elevated levels of circulating FRα as compared to matched controls. Panel A shows the number of circulating folate receptor (FRs) extrapolated from the microfiltration assay assuming a 1∶1 molar ratio of FA∶FR in both the cases and matched controls Panel B shows the densitometric signal obtained using immunoblotting. P values in panels A and B are calculated using the Wilcoxon's matched pairs test and each bar represents the mean±s.e.m. Panel C: Lane 1, immunoprecipitation negative control; lane 2, negative control (MCF-7) lysate; lane 3, positive control (KB) lysate); lane 4, control serum sample; lane 5, matched ovarian cancer serum sample; lane 6, control serum sample; lane 7 matched ovarian cancer serum sample. Arrow = M_r_ 38000 protein; KD = kilodalton. Panel D shows the linear regression analysis correlating the microfiltration binding assay data and the immunoblotting data. Each data point represents a unique individual. Open symbols represent the healthy controls and closed symbols are patients.

**Figure 3:**
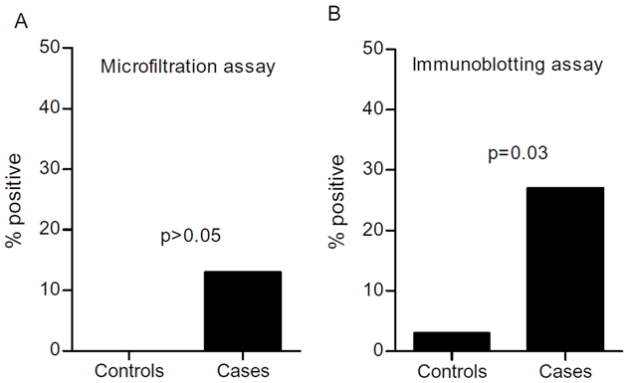
Immunoblotting may have a better positive predictive value than microfiltration assays. Panel A and B show the percentage of cases and controls that were considered positive by microfiltration or immunoblotting assay, respectively, with cutoffs established as the mean plus two standard deviations of the control values. The p-value was calculated using the Fisher's Exact Test.

Patient (i.e. cases) characteristics are detailed in Table 1. Microfilter binding assays and immunoblotting revealed that sera derived from the cases contained significantly higher mean levels of FRα relative to the controls (**Figure 2A–C**). Linear regression analysis of all the measurements from both cases and controls showed that there was a significant correlation between the results of the microfiltration and immunoblot assays (**Figure 2D**). To assess which assay may potentially be more amenable to development as a biomarker test, cutoffs were established from the control group values for both assays using the mean and two standard deviations. With this strategy, greater than 95% of control values fell below the cutoff. As shown in **Figure 3**, using this strategy, 13% and 27% of cases were considered to have elevated levels of FRα in the microfiltration and immunoblotting assays, respectively. Statistical analysis using Fisher's Exact Test showed that the fraction of cases with elevated levels of FRα, as detected with the immunoblotting assay, was significantly different than the fraction of controls considered positive. In contrast, the fraction of cases detected with the microfiltration assay was not significant, suggesting that the immunoblotting assay may potentially have a better positive predictive value.

### Levels of circulating FRα are comparable between early and advanced disease

**Figure 4:**
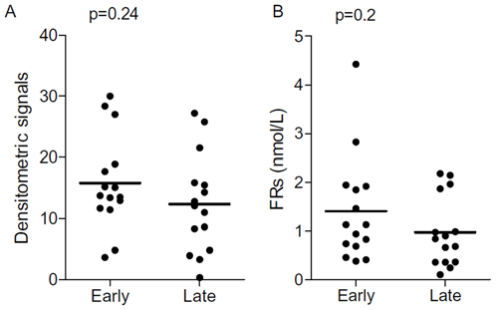
Levels of circulating FRα are similar in early and late stage ovarian cancer patients. Panels A and B shows the comparison between early and late stage densitometric and microfiltration assay signals, respectively. Each data point represents a unique individual and the horizontal line represents the mean. p values shown were calculated using a Student's T test.

We tested if there was any correlation between the levels of circulating FRα and a variety of clinical features, as well as FRα expression within the tumor. We found that circulating FRα levels (as assessed immunoblotting) were comparable between early (Stages I/II, n = 15, 16±2 au) and advanced stage (Stages III/IV, n = 15, 12±2 au, p = 0.24) patients as shown in **Figures 4A**. The differences in circulating FRα between early and advanced stage patients as assessed by binding assays was also not significant (1.4±0.3 vs. 1.0±0.2. p<0.2, **Figure 4B**). Importantly, levels of FRα in early stage patients were significantly higher than all controls (n = 30, 7±0.9 au, immunoblotting, p<0.0001 and 0.6±0.1 nmols/l binding, p = 0.02).

No significant correlations between circulating FRα and either age, grade, recurrence status or extent of debulking (optimal vs. sub-optimal) were observed (p>0.05). Tumors from 27 of 30 patients had high FRα expression, consistent with prior work, and therefore, our sample size was not large enough to determine if tumor FRα expression was associated with levels of FRα in the blood. However, of the three tumors (1 serous borderline and 2 mucinous borderline) with low tumor-associated FRα, only 1 mucinous borderline demonstrated low circulating FRα levels. This suggests that circulating FRα may not correlate well with tumor levels. Indeed, in our prior studies, we observed some discrepancies in FRα staining different tumor foci (recurrent and metastatic lesions) [14].

## Discussion

Ovarian cancer often eludes detection because of the lack of definitive early symptoms. Hence, ovarian cancer presents at advanced stage in ∼70% of patients. The biomarkers in use today are not adequate for detection of early stage cancer. Therefore, it is not surprising that ovarian cancer is the number one cause of death among all of the gynecological malignancies in recent years. Currently available markers CA-125 and proteomic profiles lack sensitivity, specificity and/or reproducibility. There is clear need for diagnostic markers to detect ovarian cancer at early stages. A molecule expressed nearly exclusively in pathogenic tissue would make an ideal biomarker and one such promising candidate is folate receptor (FR-α). FR-α is highly overexpressed in the vast majority of ovarian cancers but exhibits limited expression in tissues that are responsible for whole body retention of folates (e.g., the placenta, choroid plexus and kidney). In the current study, we tested whether it was feasible to detect circulating FRα in ovarian cancer patients and whether the circulating FRα was full-length and functional.

Our data show that patients with ovarian cancer have elevated levels of FRα relative to healthy controls. These results suggest that circulating FRα could potentially be developed as a biomarker. The increased levels of FRα in early stage disease indicates that upregulation of FRα may occur early in ovarian carcinogenesis in some patients. Indeed, Toffoli and colleagues found that nearly 79% of patients with early stage (I and II) ovarian cancer overexpressed FRα, which was essentially identical to the proportion of advanced stage patients that had FRα expression [9]. We observed in our studies that levels of circulating FRα in early stage patients was not different than advanced stage patients. However, Toffoli and colleagues found that while the proportion was similar, the levels of FRα on the tumor were significantly higher in the advanced stage patients. The lack of correlation may indicate that there are other factors regulating serum levels. For example, Mantovani and colleagues found that normal human urine samples were strongly positive for FRα immune reactivity, suggesting that there is a mechanism for its rapid kidney clearance [15]. Since the proximal tubules of the kidney strongly express the protein, one might conclude that the kidney has a well developed strategy to eliminate FRα to prevent it from accumulating in the blood and preventing FA uptake into tissues [19]. This is supported in kinetic murine modeling studies which have shown that as the tumor progresses, rise in urine levels of FRα precede sera levels considerably [15].

Intact FRα is a 38 kDa protein and in our immunoblot assay, anti-MOv18/ZEL antibody detected a protein at the same molecular weight. Furthermore, we showed that the microfiltration assay detected functional protein, which taken together suggests that FRα shed by ovarian cancer is intact and functional. The presence of FRα in the circulation of patients has therapeutic significance as a number of therapies have been developed that target cell surface FRα in cancer patients [20]. These circulating FRs may bind and interfere with clinical efficacy. This might explain high failure rates observed in some FRα-directed immunotherapies in patients with ovarian cancer. For example, Kershaw and colleagues developed an approach in which autologous T cells from patients were gene-modified with a chimeric receptor that contained a FRα-specific single chain antibody coupled to the Fc receptor gamma chain [21]. While the cells secreted IFN-γ in response to FRα, they failed to localize to tumor site, a failure that may be explained by circulating FRα which saturated the chimeric receptors. Therefore, analysis of circulating FRα in cancer patients might help in determining levels of anti-FRα targeted chimeric receptors needed for successful therapy. In addition, we also found that elevated circulating FRα was functional, suggesting potential interference with a number of folic acid-conjugated drug therapies that have been developed that require a functional binding site [20], One of the difficulties, however, in measuring serum levels of FRα is the lack of a quality assay. While our binding assays were able to detect a difference between cases and controls, the results obtained suggest that immunoblotting assay may be potentially more useful in detecting elevated levels. However, like with any blotting assay, the utility is limited by its technical complexity (i.e. immunoprecipitation followed by immunoblotting) and the results with this assay are only semi-quantitative.

In conclusion, our results demonstrate that ovarian cancer patients, including those with early disease, have elevated levels of functional intact FRα as compared to healthy controls. With further development, including larger early stage ovarian cancer sample sizes and improved assay performance, the use of FRα as a biomarker for ovarian cancer may be feasible.
